# The impact of SARS-CoV2 vaccines on the incidence of graft versus host disease in allogeneic hematopoietic stem cell transplant recipients: a single-center retrospective study

**DOI:** 10.1186/s13287-023-03326-3

**Published:** 2023-04-18

**Authors:** Dat Ngo, Jason Chen, Jose Tinajero, Ahmed Aribi, Shukaib Arslan, Guido Marcucci, Ryotaro Nakamura, Monzr M. Al Malki, Stephen J. Forman, Sanjeet Dadwal, Haris Ali

**Affiliations:** 1 Department of Pharmacy, City of Hope Medical Center, 1500 E. Duarte Road, Duarte, CA 91010 USA; 2 Department of Hematology and Hematopoietic Cell Transplantation, City of Hope, Duarte, CA USA; 3 Department of Infectious Diseases, City of Hope, Duarte, CA USA

**Keywords:** Severe acute respiratory syndrome coronavirus-2 (SARS-CoV2), COVID-19 vaccine, BNT162b2, mRNA-1273, Allogeneic hematopoietic stem cell transplantation, Graft-versus-host disease

## Abstract

This study reports the incidence of chronic graft versus host disease (GvHD) in allogeneic hematopoietic stem cell transplant (alloHCT) recipients who received SARS-CoV2 vaccination. The overall rates of new and worsening chronic GvHD combined were 14%, with median time from vaccination to GVHD being approximately three to four weeks. A majority of the cases were of mild to moderate severity and primarily localized to either the skin, mouth, or joints. Prior chronic GVHD and recent transplant were associated with higher GVHD rates following COVID-19 vaccination. More prospective studies are needed to provide a definitive mechanism for the impact of SARS-CoV2 vaccination on alloHCT patients.

In the United States, there have been two vaccines approved by the Food and Drug Administration for severe acute respiratory syndrome coronavirus-2(SARS-CoV2): Pfizer-BioNTech (New York, NY: BNT162b2) and Moderna (Cambridge, MA: mRNA-1273). Both vaccines have displayed high efficacy and safety in the general population, but there are doubts regarding the efficacy of both these vaccines in patients with cancer as this population was excluded from major clinical trials [1]. There have been recent studies published that have reported that approximately 20-30% of patients who had previously undergone an allogeneic hematopoietic cell transplantation (alloHCT) do not achieve seropositivity with these vaccines [2]. The lack of response to the vaccine could be attributed to the underlying disease and its prior treatment, the use of B- and T-cell depleting agents, use of immunosuppression for graft-versus-host disease (GvHD), and slow immune reconstitution after alloHCT. Vaccination against SARS-CoV2 continues to be strongly recommended even with a possibility of a suboptimal response because alloHCT patients suffer from high morbidity and mortality associated with infection [3]. With the landscape of SARS-CoV2 always evolving as different variants of the virus are introduced into the general population, the number and frequency of booster doses needed to continue to provide protection against SARS-CoV2 is up for debate as it is known that vaccine effect wanes over time [4, 5]. With each dose of the SARS-CoV2 vaccine administered in alloHCT patients, those who elicit an immune response to the vaccine have the added risk that the immune activation generated could lead to a flare of chronic GvHD. We recently reported in 113 alloHCT patients that 13% of patients experienced either new or worsening of GvHD symptoms after the vaccine was given [6]. Another study published found that in 34 patients with pre-existing chronic GvHD requiring treatment, the incidence of exacerbation was 26.5% [7]. Here we report an updated analysis of our experience with SARS-CoV2 vaccination and incidence of chronic GvHD in alloHCT recipients.

We retrospectively reviewed 298 adult patients who received either the Pfizer-BioNTech mRNA COVID-19 vaccine (BNT162b2) or Moderna mRNA COVID-vaccine (mRNA-1273) from December 2020 and July 2021. Inclusion criteria included patients 18 years of age or older and who had received at least two doses of vaccine in the post-HCT setting. Primary endpoint was the incidence of new and worsening GvHD based on physician documentation in the electronic medical record. New GvHD was defined as development of GvHD in a location that was not previously documented and worsening GvHD was defined as either increased severity of GvHD or requiring adjustment in GvHD regimen to address symptoms. Incidence of GvHD for the first dose was based on any reports of GvHD prior to the second dose. For subsequent doses, incidence of GvHD was reported up to 42 days after the dose was received. The severity of GvHD was independently assessed by a physician using the National Institutes of Health consensus criteria for classification of chronic GvHD [8]. Secondary endpoint was incidence of GvHD based on time vaccine was received relative to transplant date, prior systemic immunosuppressant use, and presence of GvHD prior to the vaccine. Dichotomous/categorical variables and continuous variables were analyzed using the Fisher’s exact test (using 2 × 2 or 2 × 3 contingency tables) and student’s t-test, respectively. *P* values of < 0.05 were considered statistically significant. A multivariate logistic regression was performed to identify patient characteristics and secondary endpoints associated with the incidence of GVHD. All statistical analyses were performed on RStudio, version 1.2 (RStudio Corp.,Boston, MA, USC) and *p* values < 0.05 were considered significant. Baseline characteristics are summarized in Table 1. Most of our patients received the BNT162b2 vaccine (63.1%) and were on systemic immunosuppressants at the time of vaccination (62.8%). Based on 187 patients on immunosuppressants, 17.4% were on corticosteroids. Forty percent of the patients had chronic GvHD prior to the vaccine, with patients predominantly experiencing symptoms on the skin/joints (24.8%), eyes (12.4%), and mouth (15.1%).

**Table 1:** Baseline characteristics of 298 patients who received either BNT162b2 or mRNA-1273

Characteristic	Value
Median Age, yrs (range)	61 yrs (21–79)
Gender, *n* (%)	
Male	169 (56.7)
Female	129 (40.3)
Vaccine Received, *n* (%)	
BNT162b2	188 (63.1)
mRNA-1273	110 (36.9)
Booster Received, *n* (%)	54 (18.1)
Median time to Booster, days (range)	186 days (53–266)
Primary diagnosis at HCT, *n* (%)	
AML	118 (39.6)
ALL	54 (18.1)
Myelodysplastic syndromes/ myeloproliferative neoplasms	38 (12.8)
Myelofibrosis	30 (10.1)
Other	58 (19.5)
HCT Donor, *n* (%)	
MRD	83 (27.9)
MUD	147 (49.3)
MMUD	16 (5.4)
HAPLO	48 (16.1)
MMRD	1 (0.3)
CORD	3 (1.0)
Median day after HCT, days (range)	628.5 days (71–11,199)
GvHD Prior to Vaccine, *n* (%)	119 (39.9)
Location of GvHD, *n* (%)	
Skin/Joints	74 (24.8)
Eyes	37 (12.4)
Mouth	45 (15.1)
GI	16 (5.4)
Liver	22 (7.4)
Immunosuppressants, *n* (%)	187 (62.8)
Corticosteroids	52 (17.4)
Tacro	126 (42.3)
Siro	86 (28.9)
MMF	13 (4.4)
Jakafi	46 (15.4)
Other systemic agents	8 (2.7)

All our results are shown in Tables 2 and 3. Overall, our rates of new and worsening chronic GvHD combined were 14%, which was similar to the results we published previously. There was no difference in the incidence of GvHD between the two vaccines, as the rates were 15.4% for BNT162b2 and 11.8% for mRNA-1273 (*p* = 0.491). Median time to GvHD from vaccination was around three to four weeks. Approximately 75% of patients who experienced new chronic GvHD were mild to moderate in symptoms and localized to the skin/joints and mouth. Worsening of chronic GvHD did occur at a slightly lower rate than new chronic GvHD. Half of the patients had worsened to severe symptoms of GvHD mainly in the skin/joints and mouth. Five patients were considered to have no change in the severity of GvHD symptoms, but physicians had to make modifications to their regimen to try to improve their GvHD. At the time of our analysis, there were only 54 patients who had received their booster dose at a median of 186 days (range 53–266) from when they received their first dose. Physician follow-up after the booster only occurred in 81.4% of the patients at the time of our analysis with a median follow-up time 34.5 days (range 4–87) after their booster dose. When analyzing the secondary endpoints, timing of vaccine relative to transplant date did have an impact on whether the vaccine caused chronic GvHD, as the further out from transplant the less likely a GvHD flare would occur. Patients who received their SARS-CoV2 vaccine within the first 180 days of transplant had an incidence of 30.3% compared to 11.6% of patients who received their vaccine past one year after their transplant. Those who had chronic GvHD prior to receiving their vaccine had a 10% higher incidence of developing new or worsening GvHD than those who did not have chronic GvHD prior to their vaccine. The presence of prior systemic immunosuppressants did not have an impact on development or worsening of GvHD symptoms with SARS-CoV2 vaccination. A multivariate logistic regression analysis (Table 4) of selected baseline characteristics, incidence of GvHD based on prior GvHD prior to vaccine, and incidence of GvHD based on systemic immunosuppressant use prior to vaccine identified that CORD transplants was the only significant predictor of GVHD incidence (OR = 2.85; CI 1.41–5.74; *p* = 0.003). Given the small sample size, the generalization of the statistical significance remains in question and should be further validated in larger studies.

**Table 2:** Primary endpoint of GvHD outcomes after receiving SARS-CoV2 vaccination

	Overall (N = 298)	1st Dose (N = 298)	2nd Dose (*N* = 298)	Booster (*N* = 54)
New GvHD, *n* (%)	26 (8.7)	6 (2.0)	18 (6.0)	2 (3.7)
Median time to GvHD, days (range)	–	20 days (3–28)	25 days (2–41)	9.5 days (6–13)
GvHD Severity, *n* (%)				
Mild	11 (42.3)	2 (33.3)	8 (44.4)	1 (50.0)
Moderate	9 (34.6)	3 (50.0)	6 (33.3)	0 (0)
Severe	6 (23.1)	1 (16.7)	4 (22.2)	1 (50.0)
Location				
Skin/Joints	12 (46.2)	3 (50.0)	8 (44.4)	1 (50.0)
Mouth	9 (34.6)	1 (16.7)	7 (38.9)	1 (50.0)
GI	2 (7.7)	0 (0)	2 (11.1)	0 (0)
Liver	6 (23.1)	2 (33.3)	3 (16.7)	1 (50.0)
Eyes	4 (15.4)	0 (0)	4 (22.2)	0 (0)
Other	5 (19.2)	1 (16.7)	4 (22.2)	0 (0)
Worsen GvHD, *n* (%)	16 (5.3)	8 (2.7)	8 (2.7)	0 (0)
Median time to GvHD, days (range)		21.5 days (6–28)	31.5 days (4–39)	NA
GvHD Severity, *n* (%)				
Mild	3 (18.7)	0 (0)	3 (37.5)	NA
Moderate	5 (31.3)	3 (38.0)	2 (25.0)	
Severe	8 (50.0)	5 (62.0)	3 (37.5)	
Location				
Skin/Joints	12 (75.0)	7 (87.5)	5 (62.5)	NA
Mouth	5 (31.3)	1 (12.5)	4 (50.0)	
GI	3 (18.7)	2 (25.0)	1 (12.5)	
Liver	0 (0)	0 (0)	0 (0)	
Eyes	3 (18.7)	1 (12.5)	2 (25.0)	
Other	0 (0)	0 (0)	0 (0)

**Table 3:** Secondary endpoint of GvHD outcomes after receiving SARS-CoV2 vaccination

	Value	*p* value
Incidence of GvHD relative to transplant date, *n* (%)		
0–180 days	10/33 (30.3)	0.012
180–365 days	8/57 (14.0)	
> 365 days	23/208 (11.6)	
Incidence of GvHD based on prior GvHD prior to vaccine		
Yes	33/187 (17.6)	0.011
No	8/111 (7.2)	
Incidence of GvHD based on systemic immunosuppressant use prior to vaccine, *n* (%)		
Yes	20/119 (16.8)	0.213
No	21/179 (11.7)

**Table 4:** Multivariate logistic regression analysis of GVHD

Predictor	OR (CI)	*p* value
Age	0.998 (0.995–1.002)	0.66
Gender		
Male	1.205 (0.78–1.851)	0.41
Female	1.2014 (0.779–1.852)	0.55
Vaccine Received		
BNT162b2	1.365 (0.665–2.8)	0.51
mRNA-1273	1.363 (0.66–2.79)	0.63
Primary diagnosis at HCT, *n* (%)		
AML	0.82 (0.33–2.019)	0.72
ALL	0.78 (0.315–1.922)	0.57
Myelodysplastic syndromes/ myeloproliferative neoplasms	0.712 (0.254–1.992)	0.92
Myelofibrosis	0.92 (0.37–2.28)	0.92
Other	0.836 (0.35–2.14)	0.264
HCT Donor, *n* (%)		
MRD	1.07 (0.381–2.97)	0.32
MUD	1.065 (0.38–3.002)	0.36
MMUD	0.972 (0.384–3.002)	0.641
HAPLO	1.04 (0.371–2.918)	0.49
MMRD	0.917 (0.291–2.883)	0.87
CORD	2.85 (1.41–5.74)	0.003
Incidence of GvHD relative to transplant date		
0–180 days	1.64 (0.59–5.891)	0.9813
180–365 days	1.27 (0.59–5.907)	0.9885
> 365 days	1.33 (0.42–3.118)	0.965
GvHD prior to vaccine		
Yes	1.02 (0.98–1.07)	0.166
Incidence of GvHD based on systemic immunosuppressant use prior to vaccine		
Yes	0.99 (0.71–1.38)	0.967

Our study showed that the rates of new and exacerbation of cGVHD were different in patients with a recent transplant compared to patients who had the vaccine later in the course of the alloHCT. This may help clinicians determine the risk and perhaps consider delaying immunosuppression tapering by 4–8 weeks after the vaccine if clinically feasible to prevent new or exacerbation of chronic GVHD. In conclusion, providers should continue to recommend that alloHCT patients get their SAR-CoV2 vaccination to help protect against infection but should be aware that increased monitoring or more conservative management of the GvHD regimen is necessary to ensure early recognition of new or worsening chronic GvHD.

## Data Availability

All data generated or analysed during this study are included in this published article.

## Abbreviations

SARS-CoV2: Severe acute respiratory syndrome coronavirus-2
BNT162b2: Pfizer-BioNTech mRNA COVID-19 vaccine
mRNA-1273: Moderna mRNA COVID-19 vaccine
AlloHCT: Allogeneic hematopoietic cell transplantation
HCT: Hematopoietic cell transplantation
GvHD: Graft-versus-host disease
cGvHD: Chronic graft-versus-host disease
AML: Acute myeloid leukemia
ALL: Acute lymphoid leukemia
CI: Confidence interval
MRD: Matched related donor
MUD: Matched unrelated donor
MMUD: Mismatched unrelated donor
HAPLO: Haploidentical donor
MMRD: Mismatched related donor
GI: Gastrointestinal
Tacro: Tacrolimus
Siro: Sirolimus
MMF: Mycophenolate mofetil
OR: Odds ratio
